# Supplementary material for: Iterative improvement in the automatic modular design of robot swarms
Source: PeerJ Comput Sci. 2020 Dec 7;6:e322. doi: 10.7717/peerj-cs.322 (PMC7924708; doi:10.7717/peerj-cs.322)
Supplement: Supplemental Information 3 [file peerj-cs-06-322-s003.zip › argos3/doc/api/standalone/a00587_source.html]

ARGoS: plugins/simulator/entities/quadrotor\_entity.h Source File


- Main Page
- Related Pages
- Namespaces
- Classes
- Files

- File List
- File Members

# plugins/simulator/entities/quadrotor\_entity.h

Go to the documentation of this file.

```
00001 
00007 #ifndef QUADROTOR_ENTITY_H
00008 #define QUADROTOR_ENTITY_H
00009 
00010 #include <argos3/core/simulator/entity/entity.h>
00011 #include <argos3/core/utility/math/vector3.h>
00012 
00013 namespace argos {
00014 
00015    class CQuadRotorEntity : public CEntity {
00016 
00017    public:
00018 
00019       ENABLE_VTABLE();
00020 
00021    public:
00022 
00023       enum EControlMethod {
00024          NO_CONTROL = 0,
00025          POSITION_CONTROL,
00026          SPEED_CONTROL
00027       };
00028 
00029       struct SPositionControlData {
00030          CVector3 Position;
00031          CRadians Yaw;
00032 
00033          SPositionControlData() {}
00034          SPositionControlData(const CVector3& c_position,
00035                               const CRadians& c_yaw);
00036       };
00037 
00038       struct SSpeedControlData {
00039          CVector3 Velocity;
00040          CRadians RotSpeed;
00041 
00042          SSpeedControlData() {}
00043          SSpeedControlData(const CVector3& c_velocity,
00044                            const CRadians& c_rot_speed);
00045       };
00046 
00047    public:
00048 
00049       CQuadRotorEntity(CComposableEntity* pc_parent);
00050       
00051       CQuadRotorEntity(CComposableEntity* pc_parent,
00052                        const std::string& str_id);
00053 
00054       virtual ~CQuadRotorEntity() {}
00055 
00056       virtual void Reset();
00057 
00058       EControlMethod GetControlMethod() const {
00059          return m_eControlMethod;
00060       }
00061 
00062       void SetControlMethod(EControlMethod e_control_method) {
00063          m_eControlMethod = e_control_method;
00064       }
00065 
00066       const SPositionControlData& GetPositionControlData() const {
00067          return m_sPositionControlData;
00068       }
00069 
00070       void SetPositionControlData(const SPositionControlData& s_data) {
00071          m_sPositionControlData = s_data;
00072       }
00073 
00074       const SSpeedControlData& GetSpeedControlData() const {
00075          return m_sSpeedControlData;
00076       }
00077 
00078       void SetSpeedControlData(const SSpeedControlData& s_data) {
00079          m_sSpeedControlData = s_data;
00080       }
00081 
00082       virtual std::string GetTypeDescription() const {
00083          return "quadrotor";
00084       }
00085 
00086    private:
00087 
00088       EControlMethod       m_eControlMethod;
00089       SPositionControlData m_sPositionControlData;
00090       SSpeedControlData    m_sSpeedControlData;
00091 
00092    };
00093 
00094 }
00095 
00096 #endif
```

---

Generated on 10 Jul 2018 for ARGoS by 
 1.6.1
